# Trends in use of magnetic resonance imaging and partial breast irradiation between 2011–2022 in the Netherlands: A population-based study

**DOI:** 10.1016/j.ctro.2025.101039

**Published:** 2025-08-31

**Authors:** Y.A. Civil, A.H. Eijkelboom, A.E. Veldink, M.C. van Maaren, J.H. Maduro, K.M. Duvivier, S. Siesling, H.J.G.D. van den Bongard

**Affiliations:** a Amsterdam UMC location Vrije Universiteit Amsterdam, Department of Radiation Oncology, Amsterdam, the Netherlands; b Cancer Center Amsterdam, Cancer Treatment and Quality of Life / Cancer Biology and Immunology, Amsterdam, the Netherlands; c Netherlands Comprehensive Cancer Organisation (IKNL), Department of Research and Development, Utrecht, the Netherlands; d University of Twente, Technical Medical Centre, Department of Health Technology and Services Research, Enschede, the Netherlands; e University of Groningen, University Medical Center Groningen, Department of Radiation Oncology, Groningen, the Netherlands; f Amsterdam UMC location Vrije Universiteit Amsterdam, Department of Radiology, Amsterdam, the Netherlands

**Keywords:** MRI, Partial breast irradiation, Invasive breast cancer, National trends

## Abstract

**Purpose:**

This study aimed to investigate whether the introduction of partial breast irradiation (PBI) was associated with increased MRI use in the Netherlands from 2011 to 2022, and examined the impact of MRI on treatment and outcomes.

**Methods:**

Women aged ≥50 who underwent surgery (without preoperative systemic therapy) for cT1-2N0M0 breast cancer or DCIS between 2011 and 2022 were selected from the Netherlands Cancer Registry. Logistic regression was used to analyze associations between MRI and treatment or surgical radicality, stratified by tumour type. Subgroup analyses were performed in patients meeting ASTRO PBI criteria (unifocal cT1, grade 1/2, ER-positive, pure DCIS ≤ 2,5 cm).

**Results:**

Among 119,768 patients, 35,863 (30 %) received MRI, increasing from 24 % (2011) to 37 % (2022). PBI use increased from 3 % (2017) to 21 % (2022). MRI was not associated with the probability of receiving PBI (OR 0.98, 95 % CI:0.90–1.07). In patients with invasive breast cancer, MRI was associated with fewer DCIS-involved margins (OR 0.81, 95 % CI:0.74–0.89), but a higher probability of mastectomy (OR 1.29, 95 % CI:1.24–1.34). In patients with an ASTRO-defined PBI indication, MRI use was associated with a decreased likelihood of receiving PBI in invasive cancer (OR:0.66, 95 % CI:0.60–0.72) and DCIS (OR:0.80, 95 % CI:0.75–0.85).

**Conclusion:**

This study shows rising trends in PBI and MRI use in the Netherlands from 2011 to 2022. MRI reduced PBI eligibility and enhanced surgical precision by less involved margins in patients with cT1N0 breast cancer without PST.

## Highlights

• MRI and PBI use both increased, while the combination of both remained proportionally stable.
• MRI might reduce PBI likelihood in invasive breast cancer and DCIS patients.
• MRI use is linked to higher mastectomy rates, and lower re-excision rates.
• Lobular, cTis/cT2, multifocal tumours, and in-/extra-tumoral DCIS increased MRI use.

## 1 Introduction

Most early-stage breast cancer patients are treated with breast-conserving surgery (BCS) followed by radiation therapy (RT) [1,2]. Until 2017, postoperative RT consisted of whole breast irradiation (WBI). Since then, Dutch guidelines have incorporated partial breast irradiation (PBI) as an alternative for patients deemed to be at a low risk for recurrence. These include patients aged over 50 years, diagnosed with small (≤2 cm), unifocal, estrogen-receptor (ER) positive, grade 1 or 2 non-lobular invasive carcinoma or pure DCIS ≤2.5 cm and resection margin of at least 2 mm according to the American Society for Radiation Oncology (ASTRO)recommendations [3].

The principal concern with PBI is the risk of undertreatment of occult multifocal disease after BCS, potentially increasing ipsilateral recurrence compared to WBI [4]. Therefore, careful patient selection is crucial. Previous research has shown that MRI is the most sensitive modality for detecting multifocality and determining tumour size, two crucial criteria for PBI eligibility [[5], [6], [7], [8], [9], [10]]. In 2010, the EUSOMA working group recommended preoperative MRI for patients eligible for PBI based on conventional imaging [11]. A systematic review of six studies reported that 11 % of the patients had more extensive disease detected on MRI, leading to ineligibility for PBI [12]. Similarly, in the ABLATIVE trial, prospectively investigating pathologic tumour response following single-dose preoperative PBI and BCS after six to eight months, 18 out of 31 (58 %) excluded patients were disqualified due to additional findings on MRI after mammography and ultrasound [13].

Still, MRI is not a routine assessment tool for evaluating PBI eligibility. The European Society of Medical Oncology (ESMO) guidelines recommend preoperative MRI primarily for cases with uncertainties after conventional imaging, presence of germline mutation or other high-risk pathogenic variants, lobular breast cancer, suspected multifocality or in patients with breast implants. To date, no population-based data on MRI and PBI utilization rates exist. Our retrospective population-based study aims to investigate whether the introduction of PBI was associated with increased MRI use from 2011 to 2022 in patients with early-stage breast cancer and DCIS. Additionally, the study will evaluate the impact of MRI on RT and surgical treatment strategies and outcomes.

## 2 Methods

### 2.1 Data collection

All female patients aged 50 years and older treated with surgery for cT1-2N0M0 breast cancer or DCIS in the Netherlands between 2011 and 2022 were included in this retrospective study. Patients who received preoperative systemic therapy (PST) or RT were excluded.

Data were obtained from the Netherlands Cancer Registry (NCR), a nationwide population-based registry that records patient-, tumour, and treatment- characteristics of all newly diagnosed malignancies notified by the Dutch Nationwide Pathology Databank (Palga) [14,15]. Data in the registry is manually collected by trained registrars from the Netherlands Comprehensive Cancer Organization (IKNL). Permission to use these data was obtained from the privacy board of the IKNL. According to national guidelines, preoperative MRI was indicated for patients with invasive lobular breast cancer, breast density C and D, and in cases where discrepancies between physical examination and conventional imaging, or between mammography and ultrasound may impact surgical decision-making [16]. Preoperative MRI was considered for young patients, HER2-positive tumours, or grade 3 DCIS in case of uncertainty regarding the extent or the presence of microinvasion. Data did not indicate whether these variables were collected before MRI was performed. For staging, the 7th edition of the TNM classification was used from 2011 to 2016, and the 8th edition was used from 2017 onwards [17,18]. To analyze trends in PBI use, data from 2017 to 2022 were used, reflecting the implementation of PBI use in the Dutch guidelines since 2017 [16].

### 2.2 Definitions

Hospitals were categorized as general, top clinical, or university medical centers. Tumour margins for invasive carcinoma or DCIS were classified as free, focal positive (involving ≤4 mm of the surgical margin), or more than focal positive (involving >4 mm of the surgical margin) according to the Dutch guidelines [16]. Breast cancer subtypes were defined by receptor status as 1) ER- or progesterone-receptor (PR) positive, HER2-negative, 2) HER2-positive and 3) triple negative (ER/PR/HER2 negative). Time to death or last contact with the oncological department was retrieved via regular linkage of the NCR to the Municipal Personal Records Database, which was complete until February 2024.

### 2.3 Statistical analysis

Descriptive statistics were used to summarize patient-, tumour-, and treatment-related characteristics, stratified by MRI utilization. Pearson’s Chi-square and Wilcoxon ranked sum tests were used to compare groups. Missing data were handled with multiple imputation using the R package MICE (version 3.16.0), with 25 imputations and 10 iterations [19]. The percentage of missing data ranged from 0 % to 8.2 % across variables. Logistic regression was applied to impute binary outcomes, while continuous variables were imputed with predictive mean matching, proportional odds logistic regression was used for ordered categorical variables and multinomial logistic regression for unordered categorical variables. Estimates were pooled using Rubin’s rules [20]. The impact of MRI on treatment type or margin status was assessed using multivariable logistic regression, stratified by tumour type. The models were adjusted for potential confounding variables including age, previous ipsilateral breast cancer, detection at screening, multifocality, morphology, cT stage, pN stage, molecular subtype, differentiation grade, intra-/extratumoural DCIS, most extensive surgery, and radicality invasive tumour last surgery. These variables were selected based on expert opinion, as they are key factors influencing treatment selection in clinical practice [16]. Additionally, hospital type was included as it has been previously shown to be associated with variations in treatment patterns [21]. Cramér’s V statistic was used to identify correlations between covariables. A value between 0.5 and 1.0 was considered indicative of a strong association, which led to selection of one of the two variables for inclusion in the multivariable analysis [22]. Subgroup analyses were performed to assess the association between MRI and PBI among patients treated in 2017 or later who met the ASTRO criteria for PBI eligibility, as these are the only patients eligible for PBI in the Netherlands [3]. For invasive breast cancer, the analyses included patients with unifocal, ER-positive, cT1 tumours classified as grade 1 or 2. For DCIS, the analysis focused on patients with unifocal, ≤2.5 cm, grade 1 or 2 DCIS.

Overall survival was estimated with the Kaplan-Meier method in patients with invasive breast cancer treated with PBI. The 3-year overall survival after PBI treatment was not estimated for DCIS patients, as the median survival was shorter than three years. Patients were censored at the last date of observation (February 2024) if no event had occurred. All analyses were performed in RStudio (version 4.3.2.). P-values were calculated based on available data, excluding unknown values. P-values < 0.05 were considered to be significant.

## 3 Results

From 2011 to 2022, 119,768 women with cT1-2 N0 breast cancer or DCIS underwent surgery in the Netherlands. Among them, 35,863 (29.9 %) received MRI (Table 1). The MRI group was slightly younger (63 vs. 66 years), and had more patients with larger clinical tumours (cT2) (26.3 % vs. 15.7 %), grade 2 tumours (52.6 % vs. 44.4 %), invasive lobular carcinoma (27.5 % vs. 6.1 %), and multifocal cancer (20.4 % vs. 7.7 %). MRI patients were more likely to undergo mastectomy, than no-MRI patients (37.8 % vs. 24.9 %, Table 2). Patient and tumour characteristics of patients who received PBI vs. no PBI are summarized in Table A.1.

**Table 1:** Patient and tumour characteristics for patients based on MRI utilization. ER estrogen, PR progesterone. *Only patients with invasive breast cancer.

		MRI		No MRI		p-value
No. Patients	119,768	35,863	29.9 %	83,905	70.1 %	
Age at diagnosis	Median (IQR)	63	(55–69)	66	(59–72)	<0.001
Age at diagnosis	50–59	14,043	39.2 %	23,135	27.6 %	<0.001
	60–69	12,980	36.2 %	30,304	36.1 %	
	70–79	7,765	21.7 %	22,566	26.9 %	
	>80	1,075	3.0 %	7,900	9.4 %	
Detected by screening	Yes	18,160	50.6 %	46,580	55.5 %	<0.001
	No	17,691	49.3 %	35,222	42.0 %	
	Unknown	12	0.0 %	2,103	2.5 %	
Lateralization	Left	18,208	50.8 %	42,976	51.2 %	0.155
	Right	17,655	49.2 %	40,927	48.8 %	
	Unknown	0	0.0 %	2	0.0 %	
ER status*	Positive	28,070	92.0 %	60,032	88.1 %	<0.001
	Negative	2,167	7.1 %	7,344	10.8 %	
	Unknown	266	0.9 %	757	1.1 %	
Molecular subtype*	ER/PR-positive/HER2-negative	26,269	86.1 %	55,161	81.0 %	<0.001
	HER2-positive	2,110	6.9 %	5,317	7.8 %	
	Triple negative	1,457	4.8 %	5,331	7.8 %	
	Unknown	667	2.2 %	2,324	3.4 %	
Previous ipsilateral tumour	No	35,126	97.9 %	81,933	97.6 %	0.002
	Yes	737	2.1 %	1,972	2.4 %	
Previous ipsilateral breast surgery for breast cancer	No	35,219	98.2 %	82,062	97.8 %	<0.001
	Yes	644	1.8 %	1,843	2.2 %	
Previous ipsilateral breast radiation therapy	No	35,371	98.6 %	82,331	98.1 %	<0.001
	Yes	492	1.4 %	1,574	1.9 %	
cT	is	6,298	18.1 %	17,341	20.7 %	<0.001
	1	19,966	55.7 %	52,805	62.9 %	
	2	9,415	26.3 %	13,194	15.7 %	
	Unknown	184	0.5 %	565	0.7 %	
pT	0	21	0.1 %	23	0.0 %	<0.001
	is	5,303	14.8 %	15,570	18.6 %	
	1	21,792	60.8 %	52,278	62.3 %	
	2	7,981	22.3 %	15,057	17.9 %	
	3	508	1.4 %	488	0.6 %	
	4	30	0.1 %	77	0.1 %	
	Unknown	228	0.6 %	412	0.5 %	
pN	0 (i-/i + )	27,930	77.9 %	64.738	77.2 %	<0.001
	1	5,986	16.7 %	11,659	13.9 %	
	2	262	0.7 %	446	0.7 %	
	3	79	0.2 %	155	0.2 %	
	Unknown	1,606	4.5 %	6,907	8.2 %	
Histological grade	1	8,470	23.6 %	23,681	28.2 %	<0.001
	2	18,860	52.6 %	37,257	44.4 %	
	3	7,539	21.0 %	20,428	24.3 %	
	Unknown	994	2.8 %	2,539	3.0 %	
Histology	DCIS	5360	14.9 %	15,772	18.8 %	<0.001
	Ductal	18,288	60.0 %	55,800	81.9 %	
	Lobular	8,375	27.5 %	4,165	6.1 %	
	Other	3,840	12.6 %	8,168	12.0 %	
Multifocality	Yes	7,328	20.4 %	6,436	7.7 %	<0.001
	No	28,420	79.2 %	76,954	91.7 %	
	Unknown	115	0.3 %	515	0.6 %	
Intra-/extratumoural DCIS*	Yes	16,041	52.6 %	33,703	49.5 %	<0.001
	No	14,440	47.2 %	32,838	48.2 %	
	Unknown	22	0.1 %	1,591	2.3 %	
Type of hospital	General	14,069	39.2 %	34,774	41.4 %	<0.001
	Top clinical	19,557	54.5 %	43,237	51.5 %	
	University medical center	2,237	6.2 %	5,888	7.0 %	
	Unknown	0	0.0 %	6	0.0 %

**Table 2:** Treatment characteristics of the patients stratified by MRI utilization. *Only patients with invasive breast cancer.^Margins are defined as focal positive when ≤ 4 mm of the margin is involved with tumour cells, or more than focal positive when > 4 mm is involved. ^¥^Patients could have received multiple adjuvant systemic therapies.

		MRI		No MRI		p-value
No. Patients	119,768	35,863	29.9 %	83,905	70.1 %	
Most extensive surgery	Lumpectomy	22,267	62.1 %	62,961	75.0 %	<0.001
	Mastectomy	13,573	37.8 %	20,871	24.9 %	
	Unknown	23	0.1 %	73	0.1 %	
Surgical margins invasive tumour last surgery*^	Free	28,863	94.6 %	64,516	94.7 %	<0.001
	Focal positive	1,319	4.3 %	2,502	3.7 %	
	More than focal positive	137	0.4 %	220	0.3 %	
	Not applicable	41	0.1 %	64	0.1 %	
	Unknown	143	0.5 %	831	1.2 %	
Surgical margins DCIS last surgery	Free	19,592	54.6 %	46,062	54.9 %	<0.001
	Focal positive	955	2.7 %	2,887	3.4 %	
	More than focal positive	105	0.3 %	314	0.4 %	
	Not applicable	14,431	40.2 %	31,914	38.0 %	
	Unknown	780	2.2 %	2,728	3.3 %	
Re-excision	Yes	2,156	6.0 %	4,557	5.4 %	<0.001
	No	33,707	94.0 %	79,348	94.6 %	
Lumpectomy	WBI +/- boost	17,047	47.5 %	44,660	53.2 %	<0.001
and radiation therapy	Locoregional +/- boost	2,183	6.1 %	5,341	6.4 %	
	Partial breast	1,110	3.1 %	3,999	4.8 %	
	Other	79	0.2 %	405	0.5 %	
	No	1,480	4.1 %	6,834	8.1 %	
	Unknown	368	1.0 %	1,722	2.1 %	
Mastectomy	Chestwall +/- boost	566	1.6 %	667	2.5 %	0.001
and radiation therapy	Locoregional +/- boost	1,344	3.7 %	1,472	0.8 %	
	Other	313	0.9 %	256	0.3 %	
	No	11,301	31.5 %	18,406	21.9 %	
	Unknown	49	0.1 %	70	0.1 %	
Adjuvant systemic therapy^¥^	Chemotherapy	6,340	17.7 %	11,343	13.5 %	<0.001
	Endocrine therapy	16,949	47.3 %	30,155	35.9 %	<0.001
	Targeted therapy	1,428	4.0 %	2,981	3.6 %	<0.001

**Fig. 1:**
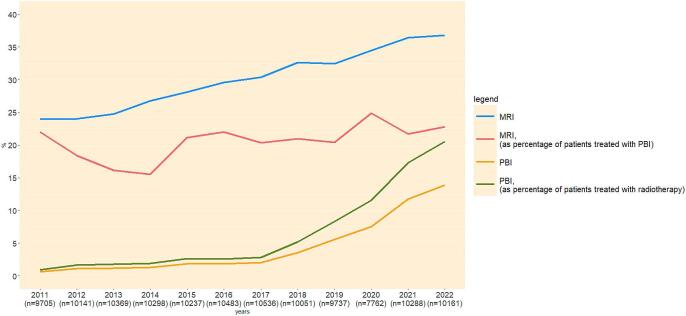
Trends of MRI and PBI utilization among breast cancer patients (2011–2022).

MRI use increased from 23.9 in 2011 to 36.8 % in 2022 (Fig. 1). The proportion of patients receiving PBI, increased from 2.0 % in 2017 to 13.9 % in 2022, with PBI among RT patients rising from 2.8 % in 2017 to 20.5 % in 2022. MRI use prior to PBI remained equal after clinical implementation of PBI (22.0 % in 2011 and 22.8 % in 2022), but the absolute number of patients receiving both MRI and PBI increased (13 out of 59 in 2011 and 322 out of 1411 in 2022) (Fig. A.1).

MRI was not associated with a higher probability of receiving PBI (OR 0.98, 95 % CI:0.90–1.07), but higher probability of receiving WBI (OR 1.16, 95 % CI:1.10–1.23) and mastectomy (OR 1.29, 95 % CI:1.24–1.34) (Table 3). MRI did not affect the radicality of invasive tumours and the probability of requiring a re-excision. However, MRI was associated with a lower probability of involved margins with the intra-/extra tumoural DCIS component (OR 0.81, 95 %CI: 0.74–0.90). Subgroup analysis of patients with unifocal cT1 tumours, classified as grade 1 or 2 and ER-positive (ASTRO criteria) showed that MRI is associated with a reduced use of PBI (OR 0.66, 95 % CI:0.60–0.72).

**Table 3:** Association between MRI utilization and probability of receiving certain treatment or (more than) focal irradical surgery in patients with invasive breast cancer. (n = 98,636) NA not applicable.

		PBI^b^	WBI	Radicality invasive tumour last surgery	Radicality DCIS last surgery	Re-excision	Mastectomy	PBI^c^
*Unadjusted*	OR	0.53	0.88	1.19	0.70	1.30	1.78	0.66
	95 % CI	0.49–0.57	0.86–0.91	1.12–1.27	0.64–0.76	1.22–1.38	1.730–1.83	0.60–0.72
*Adjusted*^a^	OR	0.98	1.16	0.93	0.81	0.93	1.29	NA
	95 % CI	0.90–1.07	1.10–1.23	0.86–1.00	0.74–0.90	0.87–1.00	1.24–1.34	

a Adjusted for age, previous ipsilateral breast cancer, detection at screening, multifocality, morphology, cT stage, pN stage, molecular subtype, differentiation grade, intra-/extratumoural DCIS (excluded in analysis of radicality DCIS last surgery), type of hospital, most extensive surgery (excluded in analysis of mastectomy), radicality invasive tumour last surgery (excluded in analysis radicality invasive tumour last surgery).

b Time period 2017–2022 (n = 48,637).

c Only patients with unifocal cT1, grade 1 or 2 and estrogen-receptor positive tumours according to ASTRO criteria (n = 26,935).

In patients with DCIS, MRI did not affect the probability of receiving PBI (OR 0.93, 95 % CI:0.69–1.26) or WBI (OR 0.95, 95 % CI:0.85–1.07), nor did it influence the likelihood of positive margins (OR 1.07, 95 % CI:0.92–1.24) (Table 4). However, MRI was associated with a reduced probability of re-excision (OR 0.74, 95 % CI:0.67–0.83) and an increased likelihood of receiving mastectomy (adjusted OR 1.85, 95 % CI:1.72–1.98). A subgroup analysis of the association between MRI and PBI in patients with unifocal ≤2.5 cm grade 1 or 2 DCIS (according to ASTRO criteria) revealed an OR of 0.80 (95 % CI:0.75–0.85).

**Table 4:** Probability of receiving certain treatment or radicality depending on MRI performance in patients with DCIS (n = 21,132). NA not applicable.

		PBI^b^	WBI	Radicality DCIS last surgery	Re-excision	Mastectomy	PBI^c^
*Unadjusted*	OR	0.51	0.67	0.92	0.87	2.21	0.61
	95 % CI	0.38–0.68	0.63–0.72	0.79–1.06	0.79–0.97	2.07–2.36	0.59–0.63
*Adjusted*^a^	OR	0.93	0.95	1.07	0.74	1.85	NA
	95 % CI	0.69–1.26	0.85–1.07	0.92–1.24	0.67–0.83	1.72–1.98	

a Adjusted for age, previous ipsilateral breast cancer, detection at screening, multifocality, differentiation grade, type of hospital, most extensive surgery (excluded in analysis of mastectomy), radicality DCIS last surgery (excluded in analysis of radicality DCIS last surgery).

b Time period 2017–2022 (n = 9,898).

c Only patients with unifocal grade 1 or 2 ductal carcinoma in situ according to ASTRO criteria (n = 3,852).

The median follow-up of-patients treated with PBI after 2017 was 3.1 years (IQR 1.4–4.6). OS at 3 years for these patients was 97.8 % (95 % CI 97.1–98.5 %) with 1,061 patients at risk at 3 years. OS was not different in the MRI and no-MRI groups (p = 0.3).

## 4 Discussion

This study showed an increase in MRI and PBI use in the Netherlands from 2011 to 2022 among patients with T1-2 N0 breast cancer or DCIS who did not receive PST. However, the combined use of MRI and PBI did not rise. MRI was associated with a lower probability of involved surgical margins with intra-/extra-tumoural DCIS-component and increased risk of mastectomy. Among patients eligible for PBI according to ASTRO criteria, MRI reduced the likelihood of receiving PBI.

Similar US and Canadian studies also reported rising MRI and PBI use [23,24]. According to a report from the National Cancer Database, the use of PBI in the US increased from 3.4 % in 2002 to 12.4 % in 2010 [24]. Despite the increasing MRI and PBI use in the Netherlands, MRI rates among patients undergoing PBI remained stable over time. Although MRI is the most sensitive modality for assessing tumour size and multifocality −key eligibility criteria for PBI- the proportion of patients undergoing MRI before PBI remains low. In our study, subgroup analysis of patients eligible for PBI according to ASTRO criteria showed that MRI reduced the probability of receiving PBI, consistent with previous data showing 11 % PBI-ineligibility post-MRI [12]. These findings may be explained by the detection of a larger-than-expected tumor size, multifocality or contralateral breast cancer. Multivariable analysis confirmed that patients were more likely to receive WBI following MRI. In the Netherlands, there are no formal MRI recommendations for PBI selection. In addition, one Dutch university medical center (UMC) with a large breast cancer population uses contrast-enhanced mammography instead of MRI, a technique gaining interest in more hospitals and potentially influencing national MRI trends. On the other hand, the updated ASTRO criteria, lowering the PBI eligibility age to 40, could increase MRI use, as MRI is often recommended in younger women [25]. Conversely, excluding BRCA mutation carriers from PBI eligibility may reduce MRI use.

Besides the impact of MRI on PBI use, it can also have a clinical impact on the other treatment modalities for breast cancer patients. The observed improvement in DCIS radicality, as opposed to invasive breast cancer may be explained by the underestimation of DCIS size on mammography in the no-MRI group, particularly in cases of high-grade DCIS [26,27]. The frequency of DCIS underestimation is greater than that of invasive breast cancer [28]. This trial defined negative tumour margins as ‘no ink on tumour’, limiting the generalizability of results to populations with different margins definitions. In 2014, Society of Surgical Oncology (SSO) and ASTRO recommended this standard, though some evidence suggests a margin 1 mm may be preferable [29,30].

A recent *meta*-analysis of nearly 136,000 patients demonstrated a benefit from MRI in decreasing re-excision rates (OR 0.63 (95 % CI 0.45–0.89) [31]. In our study, MRI use did not affect re-excision rates in patients with invasive breast cancer, despite a reduction in intra-/extra-tumoural DCIS involved margins. This finding may be partly explained by the higher probability of undergoing mastectomy, although this was adjusted for in the multivariable analysis, residual confounding cannot be ruled out. On the other hand, the lower probability of intra-/extra-tumoural DCIS involved margins could increase the use of PBI. Additionally, patient preferences and cosmetic considerations may have influenced surgical decision-making. The increased likelihood of receiving a mastectomy has also been demonstrated by various studies [[32], [33], [34], [35], [36], [37]]; however, not all studies have shown this association, making the issue controversial [31,38]. A recent *meta*-analysis indicated that mastectomy is associated with lower overall survival compared to breast-conserving therapy, raising concerns that preoperative MRI might adversely affect oncological outcomes [39]. However, as no RCTs were included and all studies were observational, significant bias cannot be excluded. An increased mastectomy rate is anticipated after MRI, as these patients in our study had less favorable characteristics and higher-risk tumours, such as larger tumours, axillary involvement, and higher grade. This association between MRI and increased mastectomy rates persisted even after adjusting for potential confounders. Identification of additional lesions or more accurate tumour sizing on MRI could be key factors influencing surgical radicality, the need for re-excision and the decision to perform a mastectomy [5,7,10,35,[40], [41], [42]].

Despite the clinical impact of MRI on surgical outcomes and RT, its long-term oncological benefit remains debated [43]. The BREAST-MRI trial, which randomized 524 patients (stage 0-III) to MRI vs. no-MRI, found no differences in local control and OS after 5.9 years, [44]. Likewise, the POMB trial (440 patients <56 years) showed no 10-year survival benefit, though combined locoregional, distant and contralateral recurrence outcomes favored MRI (p = 0.048) [45]. A *meta*-analysis by Eisen et al. also found reduced recurrence with MRI (HR = 0.77, 95 % CI = 0.65–0.90) [31]. On the other hand, the *meta*-analysis of Houssami et al. and the COMICE trial found no effect on the 8-year recurrence rates [46,47].

MRI’s potential survival benefit may stem from increased detection of contralateral breast cancer, which occurs four times more often with MRI [33]. Early detection of contralateral breast cancer might lead to a relative increase in survival [48]. Notably, MRI-based contralateral screening has shown a survival benefit, particularly in patients with tumours >2 cm and grade 3 tumours [49]. Further evidence from the ongoing ECOG-ACRIN trial (NCT01805076) will further clarify MRI’s role in HER2-positive and triple negative breast cancer.

In the Netherlands, endocrine therapy is typically given for ER-positive tumours ≥1 cm (grade 2 and 3) or ≥2 cm (grade 1) [16]. Consequently, only 48 % of our cohort received endocrine therapy, despite 89 % having ER-positive tumours, potentially influencing recurrence rates and OS. In contrast, the IMPORT-LOW trial, which randomized 2,018 patients to WBI, reduced-dose WBI and PBI, reported 90 % endocrine therapy use (95 % ER-positive), and 5-year OS of 96 %[50]. Similarly, in the Florence trial, which randomized 520 patients to PBI or WBI, had 66 % therapy use, 95 % ER-positive tumours, and 10-year OS of 91 % [51]. The ongoing EPOPE trial may clarify the value of endocrine therapy in PBI patients [52].

This study has strengths and limitations. The nationwide population-based cancer registry enhanced the generalizability of the results and provided a dataset for robust multivariable models. However, the retrospective design presents limitations. Factors not captured in the NCR, such as breast density, gene mutations, patient preferences and frailty, may have influenced MRI and PBI use. Additionally, shared decision-making between radiation oncologists and patients, based on individual values and treatment preferences, may also influence the choice between PBI and WBI. Another important limitation is the uncertainty regarding whether multifocality and the cT stage were detected prior to MRI or as a result of the MRI. Patients undergoing MRI are more likely to be ineligible for PBI based on a larger tumour size and/or multifocality on MRI, which was not visualized on mammography, while patients without MRI more often remain eligible for PBI. This introduces detection bias, and possibly overestimates the negative effect of MRI on PBI. In addition, the association between MRI use and reduced PBI may partly reflect selection bias, as MRI was less frequently performed in the older patients with screen-detected, low-grade tumors, a group more likely to receive PBI.

Furthermore, the recent introduction of PBI as a standard treatment for low-risk patients has led to a relatively short follow-up duration especially in patients with DCIS for which the survival analyses could not be performed due to short of follow-up time. Longer follow-up periods would provide more valuable insights into the overall survival in this patient group. Moreover, data on local recurrence data, if present, could provide important insights into the clinical impact of MRI use.

A recent *meta*-analysis reported a higher local recurrence rate after PBI compared to WBI (5 % vs. 3 %) [53]. Notably, recent studies on external beam PBI have not documented the use of MRI in the diagnostic workup, suggesting that undetected multifocality may contribute to the increased recurrence risk. Studies investigating preoperative PBI have used MRI to determine patient eligibility, and report local recurrence rates up to 3 % after a maximum median follow-up of 5 years [[54], [55], [56], [57]]. Most PBI-eligible patients do not meet current MRI criteria guidelines, yet MRI is crucial, considered by some authors, for assessing PBI suitability, such as unifocal disease [16]. However, the costs and limited capacity of MRI might restrict the routine use of MRI prior to PBI [47]. Recent literature suggests performing preoperative MRI only in specific cases, such as young women, those with initial cancer presenting as interval cancer, ER-negative cancer, lobular breast cancer, dense breasts, or when breast conservation without RT is being considered [58]. Overestimation of tumour size may lead to unnecessary mastectomies, and false positives could result in unneeded biopsies and imaging [6]. Still, MRI is more accurate than conventional imaging to determine tumour size and multifocal disease [41,59]. To provide more robust evidence on the role of MRI in improving outcomes for PBI patients, an RCT randomizing PBI-eligible patients to MRI or no-MRI would be ideal. However, this study design would not be ethically feasible in current clinical practice, as MRI is widely incorporated into diagnostic guidelines based on evidence in literature, making randomization to a no-MRI group ethically inappropriate.

## 5 Conclusion

This nationwide population-based study revealed increasing trends in MRI and PBI use among patients with T1-2 N0 breast cancer without PST and DCIS, along with a steady combination of MRI and PBI in the Netherlands from 2011 to 2022. MRI could be a valuable tool for selecting patients eligible for PBI, as it reduced likelihood of receiving PBI in patients meeting the PBI criteria, while increasing the likelihood of WBI. MRI enhanced surgical precision by reducing the rate of involved margins when an intra-/extra-tumoural DCIS component is present in invasive tumours, although it did not impact re-excision rates. However the effect of MRI on long-term outcomes, such as the local recurrence rates and survival after PBI, warrants further investigation through large-scale studies with extended follow-up.

## Patient consent statement

Not applicable.

## Funding

This study was not funded.

## Declaration of competing interest

The authors declare the following financial interests/personal relationships which may be considered as potential competing interests: HJGDB has received a grant from Varian, travel reimbursement from Elekta, and multiple grants from the Dutch Cancer Society. All remaining authors have declared no conflicts of interest.
