# Supplementary material for: Trends in use of magnetic resonance imaging and partial breast irradiation between 2011–2022 in the Netherlands: A population-based study
Source: Clin Transl Radiat Oncol. 2025 Aug 31;55:101039. doi: 10.1016/j.ctro.2025.101039 (PMC12444457; doi:10.1016/j.ctro.2025.101039)

**Appendix A**

**Table A.1.** Patient and tumour characteristics for patients based on PBI utilization.

**Figure A.1.** Trends in MRI utilization in relation to chest wall irradiation, PBI and WBI

**Table A.1.** Patient and tumour characteristics for patients based on PBI utilization.

|  |  | PBI |  | No PBI |  | p-value |
| --- | --- | --- | --- | --- | --- | --- |
| No. Patients | 119,768 | 5,122 | 4.3% | 114,646 | 95.7% |  |
| MRI | Yes | 1,114 | 21.7% | 34,749 | 30.3% |  |
|  | No | 4,008 | 78.3% | 79,897 | 69.7% | <0.001 |
| Age at diagnosis | Median (IQR) | 65 | 59-71 | 65 | 57-72 | 0.878 |
| Age at diagnosis | 50-59 | 1,331 | 26.0% | 35,847 | 31.3% | <0.001 |
|  | 60-69 | 2,285 | 44.6% | 40,999 | 35.8% |  |
|  | 70-79 | 1,337 | 26.1% | 28,994 | 25.3% |  |
|  | >80 | 169 | 3.3% | 8,806 | 7.7% |  |
| Detected by screening | Yes | 3,483 | 68.0% | 61,257 | 53.4% | <0.001 |
|  | No | 1,639 | 32.0% | 51,274 | 44.7% |  |
|  | Unknown | 0 | 0.0% | 2,115 | 1.8% |  |
| Lateralization | Left | 2,550 | 49.8% | 58,634 | 51.1% | 0.059 |
|  | Right | 2,572 | 50.2% | 56,010 | 48.9% |  |
|  | Unknown | 0 | 0.0% | 2 | 0.0% |  |
| ER status* | Positive | 4,692 | 98.8% | 83,410 | 88.8% | <0.001 |
|  | Negative | 48 | 1.0% | 9,463 | 10.1% |  |
|  | Unknown | 7 | 0.1% | 1,016 | 1.1% |  |
| Molecular subtype* | ER/PR-positive/HER2-negative | 4,597 | 96.8% | 76,883 | 81.8% | <0.001 |
|  | HER2-positive | 68 | 1.4% | 7,359 | 7.8% |  |
|  | Triple negative | 35 | 0.7% | 6,753 | 7.2% |  |
|  | Unknown | 47 | 1.0% | 2,944 | 3.1% |  |
| Previous ipsilateral tumour | No | 5,098 | 99.5% | 111,961 | 97.7% | <0.001 |
|  | Yes | 24 | 0.5% | 2,685 | 2.3% |  |
| Previous ipsilateral breast surgery for breast cancer | No | 5,109 | 99,7% | 112,172 | 97.8% |  |
|  | Yes | 13 | 0.3% | 2,474 | 2.2% |  |
| Previous ipsilateral breast radiation therapy | No | 5,115 | 99.9% | 112,587 | 98.2% | <0.001 |
|  | Yes | 7 | 0.1% | 2.059 | 1.8% |  |
| cT | is | 402 | 7.9% | 23,237 | 20.3% | <0.001 |
|  | 1 | 4,438 | 86.6% | 68,333 | 59.6% |  |
|  | 2 | 272 | 5.3% | 22,337 | 19.5% |  |
|  | Unknown | 10 | 2.0% | 739 | 0.6% |  |
| pT | 0 | 3 | 0.2% | 41 | 0.0% | <0.001 |
|  | is | 374 | 7.3% | 20,499 | 17.9% |  |
|  | 1 | 4,500 | 87.9% | 69,570 | 60.7% |  |
|  | 2 | 234 | 4.6% | 22,804 | 19.9% |  |
|  | 3 | 0 | 0.0% | 996 | 0.9% |  |
|  | 4 | 0 | 0.0% | 107 | 0.1% |  |
|  | Unknown | 11 | 0.2% | 629 | 0.5% |  |
| pN | 0 (i-/i+) | 4,939 | 96.4% | 87,729 | 76.5% | <0.001 |
|  | 1 | 49 | 1.0% | 17,596 | 15.3% |  |
|  | 2 | 0 | 0.0% | 708 | 0.6% |  |
|  | 3 | 0 | 0.0% | 234 | 0.2% |  |
|  | Unknown | 134 | 2.6% | 8,379 | 7.3% |  |
| Histological grade | 1 | 2,235 | 45.4% | 29,826 | 26.0% | <0.001 |
|  | 2 | 2,520 | 49.2% | 53,597 | 46.7% |  |
|  | 3 | 225 | 4.4% | 27,742 | 24.2% |  |
|  | Unknown | 52 | 1.0% | 3,481 | 3.0% |  |
| Histology | DCIS | 375 | 7.3% | 20,757 | 18.1% | <0.001 |
|  | Ductal | 4,141 | 80.8% | 69,947 | 61.0% |  |
|  | Lobular | 159 | 3.1% | 12,381 | 10.8% |  |
|  | Other | 447 | 8.7% | 11,561 | 10.1% |  |
| Multifocality | Yes | 66 | 1.3% | 13,698 | 11.9% | <0.001 |
|  | No | 5,052 | 98.6% | 100,322 | 87.5% |  |
|  | Unknown | 4 | 0.1% | 626 | 0.5% |  |
| Intra-/extratumoural DCIS* | Yes | 1,961 | 41.3% | 46,182 | 49.2% | <0.001 |
|  | No | 2,786 | 58.7% | 46,093 | 49.1% |  |
|  | Unknown | 0 | 0.0% | 1,614 | 1.7% |  |
| Type of hospital | General | 1,783 | 34.8% | 47,060 | 41.0% | <0.001 |
|  | Top clinical | 3,140 | 61.3% | 59,654 | 52.0% |  |
|  | University medical center | 199 | 3.9% | 7,926 | 6.9% |  |
|  | Unknown | 0 | 0.0% | 6 | 0.0% |  |

ER estrogen, PR progesterone. *Only patients with invasive breast cancer.

**Figure A.1.** Trends in MRI utilization in relation to PBI,WBI and post-mastectomy radiation therapy


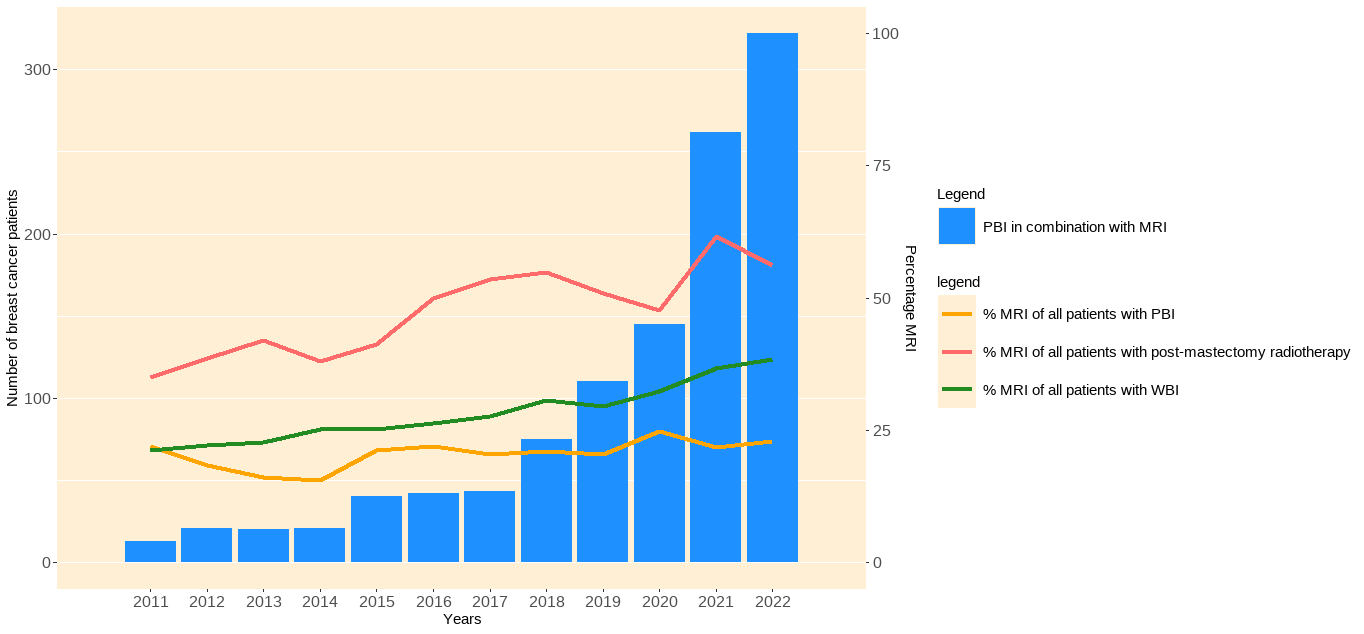

Supplement: Supplementary Data 1 [file mmc1.docx]
